# Identification of genomic loci regulating grain iron content in *aus* rice under two irrigation management systems

**DOI:** 10.1002/fes3.329

**Published:** 2021-09-30

**Authors:** Partha Talukdar, Anthony J. Travis, Mahmud Hossain, Md Rafiqul Islam, Gareth J. Norton, Adam H. Price

**Affiliations:** ^1^ School of Biological Sciences University of Aberdeen Aberdeen UK; ^2^ Department of Soil Science Bangladesh Agricultural University Mymensingh Bangladesh

**Keywords:** *aus*, gene, GWA, Iron, QTL, rice

## Abstract

Iron (Fe) deficiency is one of the common causes of anaemia in humans. Improving grain Fe in rice, therefore, could have a positive impact for humans worldwide, especially for those people who consume rice as a staple food. In this study, 225–269 accessions of the Bengal and Assam Aus Panel (BAAP) were investigated for their accumulation of grain Fe in two consecutive years in a field experiment under alternative wetting and drying (AWD) and continuous flooded (CF) irrigation. AWD reduced straw Fe by 40% and grain Fe by 5.5–13%. Genotype differences accounted for 35% of the variation in grain Fe, while genotype by irrigation interaction accounted for 12% of the variation in straw and grain Fe in year 1, with no significant interactions detected in year 2. Twelve rice accessions were identified as having high grain Fe for both years regardless of irrigation treatment, half of which were from BAAP *aus* subgroup 3 which prominently comes from Bangladesh. On average, subgroup 3 had higher grain Fe than the other four subgroups of *aus*. Genome‐wide association mapping identified 6 genomic loci controlling natural variation of grain Fe concentration in plants grown under AWD. For one QTL, nicotianamine synthase *OsNAS3* is proposed as candidate for controlling natural variation of grain Fe in rice. The BAAP contains three haplotypes of *OsNAS3* where one haplotype (detected in 31% of the individuals) increased grain Fe up to 11%. Haplotype analysis of this gene in rice suggests that the ability to detect the QTL is enhanced in the BAAP because the high Fe allele is balanced in *aus*, unlike *indica* and *japonica* subgroups.

## Graphical abstract

Genome‐wide association mapping is conducted for grain iron content on the Bengal and Assam Aus Panel of rice that has been grown in conventional flooding and alternate wetting and drying (AWD) irrigation over two years. AWD reduced shoot and grain by 40% and ~10% respectively. Six quantitative trait loci were detected including at candidate gene OsNAS3, where allelic variation in aus enables genetic detection

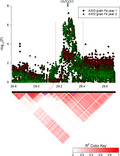

## 1 INTRODUCTION

Plants require iron (Fe) for their growth, development and to complete their life cycle (Tripathi et al., 2018). As an essential element, Fe is required by plants for a number of cellular processes such as respiration, hormone biosynthesis, chlorophyll biosynthesis, photosynthetic electron transport, nitrogen assimilation and pathogen defence (Hansch & Mendel, 2009). Fe deficiency results in poor growth, reduced yields and chlorosis in plants (Hansch & Mendel, 2009).

Fe is an essential macronutrient in the human diet required to maintain several biological processes including DNA and RNA synthesis, cell proliferation and differentiation and the mitochondrial respiratory chain (Lieu et al., 2001). Haemoglobin, an Fe containing protein, is an important molecule in oxygen carrying in blood (Chen et al., 2015). Ferritin is another Fe containing protein molecule that stores and releases Fe in a controlled fashion to maintain the level of Fe in the human body (Mesquita et al., 2020). As a result of Fe deficiency, humans can suffer several diseases (Abbaspour et al., 2014; Miller, 2013), for example anaemia and tissue hypoxia (Theurl et al., 2016).

Plants are the main dietary source of Fe for human around the world (Graham et al., 2007). As a staple and gluten‐free food, rice (*Oryza sativa* L.) is consumed by billions of people globally (Fageria, 2007; Hosseini et al., 2018). Polished rice is usually preferred than brown rice across the world. However, polishing rice results in a loss of Fe. A recent study observed the mean concentration of Fe was 63% lower in rice after it had been polished (Maganti et al., 2020). This indicates that people across the world who solely depends on rice for their staple food will consume less Fe from rice and therefore boosting rice Fe would help these people. Rice grain has a narrow genetic variation for endosperm Fe with which to try to improve dietary Fe (Glahn et al., 2002; Gregorio et al., 2000; Meng et al., 2005). To date, popular polished rice contains approximately 2 µg/g grain Fe but the targeted grain concentration is 15 µg/g (Bouis et al., 2011). Transgenic approaches were used to increase grain Fe with great success. For example, Trijatmiko et al. (2016) achieved 15 µg/g Fe in endosperm by overexpressing rice nicotianamine synthase (*OsNAS2*) and soybean ferritin. However, natural alleles for breeding are still required for those countries where genetically modified (GM) and gene editing approaches are not legally approved. Therefore, if a wider rice population is examined, and stable loci for high grain Fe and underlying gene(s) are identified, there is a scope that Fe content in rice can be increased through breeding, GM or gene editing approaches.

Several gene and enzyme families have been identified which play important roles for Fe uptake and accumulation in rice. For example, iron regulated transporter (*IRT*), yellow stripe‐like (*YSL*), nicotianamine synthesis (*NAS*), vacuole iron transporter (*VIT*) and natural resistance‐associated macrophage protein (*NRAMP*) are the well‐known gene and enzyme families to be involved in Fe trafficking in plants (Conte & Walker, 2011; Kobayashi et al., 2014). To date, two *IRTs* (*IRT1* and *IRT2*) (Eide et al., 1996; Ishimaru et al., 2006; Robinson et al., 1999; Vert et al., 2001; Wairich et al., 2019), four *YSLs* (Inoue et al., 2009; Ishimaru et al., 2010; Kakei et al., 2012; Koike et al., 2004), three *NASs* (Inoue et al., 2003; Ishimaru et al., 2006; Johnson et al., 2011; Lee et al., 2009; Takagi, 1976; Trijatmiko et al., 2016), two *VITs* (Che et al., 2021; Zhang et al., 2012) and several *NRAMPs* (Curie et al., 2000; Ishimaru et al., 2012; Peris‐peris et al., 2017; Swamy et al., 2021; Takahashi et al., 2011) have been categorised which are involved in Fe transport in rice. The *IRT* family is involved in Fe uptake and translocation in rice (Eide et al., 1996; Ishimaru et al., 2006; Robinson et al., 1999; Vert et al., 2001). *OsYSL15* (Inoue et al., 2009; Lee et al., 2009) and *OsYSL16* (Kakei et al., 2012) are involved in the influx of metal‐NA complex and efflux of Fe (II) in rice, whereas *OsYSL2* (Ishimaru et al., 2010; Koike et al., 2004) is involved in Fe translocation. The *NAS* family is involved in de‐oxymugineic acid (DMA) biosynthesis required for Fe (III)‐DMA uptake and translocation (Inoue et al., 2003; Ishimaru et al., 2006; Takagi, 1976). The *VIT* family is responsible for Fe subcellular sequestration and translocation of Fe between flag leaves and seeds (Che et al., 2021; Zhang et al., 2012). The *NRAMP* family is responsible for ferrous Fe uptake and translocation in rice (Curie et al., 2000; Ishimaru et al., 2012; Peris‐Peris et al., 2017; Swamy et al., 2021; Takahashi et al., 2011). Several genes have been also classified as Fe transporter in rice such as a mitochondrial iron transporter (Os03g0296800) (Bashir et al., 2011), a paralogue of a DNA glycosylase domain protein (*OsROS1*) (Liu et al., 2018) and a multidrug toxic compound extrusion transporter (*OsFRDL1*) (Yokosho et al., 2009, 2016).

Mechanisms controlling Fe uptake and accumulation in rice and other higher plants have been previously elucidated. It is proposed that there are two different strategies (strategy I and strategy II) used by rice to uptake Fe from soil. In strategy I, rice plants release protons into the rhizosphere to increase ferric ions (Fe^3+^) solubility (Fox & Guerinot, 1998). Subsequently, Fe^3+^ is reduced to Fe^2+^ by ferric reductase‐oxides (FRO) enzyme and Fe^2+^ ions enter root plasma membrane by IRTs (Mukherjee et al., 2006; Robinson et al., 1999; Vert et al., 2002; Wu et al., 2005). In strategy II, the biosynthesis of Fe‐chelators, known as mugineic acid family phytosiderophores (MAs), solubilises Fe in soil and produces a Fe‐phytosiderophores complex (Fe^3+^‐PS) (Bashir et al., 2006; Inoue et al., 2003; Nozoye et al., 2011). The complexes (Fe^3+^‐PS) are taken up into root cells by the transmembrane YSL proteins (Inoue et al., 2009; Ishimaru et al., 2006; Kakei et al., 2012). The relative importance of each of these strategies is likely to vary according to environmental conditions because rice is grown in diverse soil water conditions (eg. flooded, non‐flooded) which will impact the solubility and availability of Fe ions. For example, under non‐flooded condition, Fe is predominantly present in the oxidised, almost insoluble, Fe^3+^ form which may not be available to uptake by rice root (Morrissey & Guerinot, 2009). On the other hand, under flooded conditions, Fe^3+^ is reduced to Fe^2+^ which is more efficiently taken up by rice roots and has the potential to achieve toxic levels of soil Fe availability (Becker & Asch, 2005).

Previous studies have demonstrated that several quantitative trait loci (QTL) are responsible for shoot/straw and grain Fe accumulation in rice. Several genetic mapping studies using biparental crosses have been conducted to identify genomic loci regulating Fe content in rice (Anuradha et al., 2012; Calayugan et al., 2020; Dixit et al., 2019; Jeong et al., 2020; Jeong, Lee, et al., 2020; Norton et al., 2010; Swamy et al., 2018; Zhang et al., 2014). Recently, a number of genome‐wide association (GWA) mapping studies have been conducted to identify genomic loci controlling grain Fe using different rice populations (Bollinedi et al., 2020; Descalsota et al., 2018; Liu et al., 2020; Pradhan et al., 2020; Yang et al., 2018; Zaw et al., 2019). However, no GWA study has been conducted yet to examine a larger rice population for grain Fe concentration in flooded and alternative wetting and drying (AWD) conditions where genotype‐environment interactions are also considered.

AWD is a water saving technique which is currently used for rice production in some countries (Carrijo et al., 2017, 2018). Although AWD is not widely adopted by rice farmers, it is more sustainable than conventional rice farming as AWD reduces both water inputs and emits less greenhouse gases (Carrijo et al., 2017; Howell et al., 2015; Lampayan et al., 2015). Several studies have been conducted recently to understand the impact on shoot/straw and grain quality in rice using AWD system (Carrijo et al., 2018). A study conducted by Norton, Travis, et al., (2017) on 22 rice accessions observed that there is a significant reduction in straw and grain Fe when plants are cultivated under AWD compared to plants cultivated using continuously flooded (CF) field management. Since AWD is becoming more popular and adopted by farmers as a water saving technique, there is a scientific importance to evaluate the impact AWD has on grain traits.

In this study, a rice population, the Bengal and Assam Aus Panel (BAAP), was utilised to appreciate Fe accumulation in brown rice under two different water management systems (AWD, CF). The BAAP is a rice population which is mainly comprised of accessions from the *aus* subpopulation of rice (Travis et al., 2015). A study conducted by Norton et al. (2018) identified 5 different subgroups within BAAP denoting that it consists of a structured and genetically diverse population. Previous studies demonstrated that *aus* subpopulation has agriculturally important alleles for disease resistance (Garris et al., 2003), deep water and submergence tolerance (Hattori et al., 2009; Xu et al., 2006), drought tolerance and drought resistance (Bernier et al., 2009) and low phosphorous tolerance (Gamuyao et al., 2012). Therefore, the *aus* is recognised as an important source of genetic material for plant breeders to develop stress‐tolerance and climate‐resilient rice varieties (Kim et al., 2016). Recent studies on *aus* subpopulation in rice have also identified genes controlling variation of grain arsenic and cadmium accumulation and salt tolerance in rice (Chen et al., 2020; Norton et al., 2019, 2021).

To understand Fe accumulation trait in rice, this study focussed on the BAAP population which is mainly consisted of *aus* subpopulation of rice. The population was cultivated in a field experiment in Bangladesh for two consecutive years (Norton et al., 2018). The population has been genotyped with 2 million single‐nucleotide polymorphisms (SNPs) and consisted of 5 different subgroups which are utilised to perform genome‐wide association (GWA) mapping of straw and grain Fe traits. The goal of this study is to understand how AWD impacts on straw and grain Fe accumulation in rice compared to CF and identify genomic loci and underlying gene(s) controlling Fe variation in rice.

## 2 METHODS

### 2.1 Experimental materials

The rice population used was the Bengal and Assam Aus Panel (BAAP) which has been previously used to study natural variation of multiple phenotypes (Chen et al., 2020; Norton et al., 2018, 2019, 2021). The BAAP consists of 266 *aus* rice accessions including a number of high yielding accessions originating from Bangladesh and India and a number of other rice accessions including the *OryzaSNP* panel (McNally et al., 2009; Norton et al., 2018).

### 2.2 Experimental sites and procedures

The field experiments were conducted in Mymensingh, Bangladesh, for two consecutive years in the dry season of 2013 (Year 1) and 2014 (Year 2) under AWD and CF conditions. The detail information about the physical and chemical properties of soil in the experimental site (Hossain et al., 2009; Norton, Shafaei, et al., 2017), resources (eg fertiliser and water) used for the study and the method applied in field experiment have been previously reported (Norton et al., 2018). Briefly, rice seedlings were grown in the field and transplanted by hand. A total of 8 experimental plots (5 m × 11.4 m) were used (4 replicates of AWD and 4 replicates of CF) to grow plants, with two plants per hill with a distance of 20 cm between each hill in a row and a 20 cm distance between each row of 2 m length. Rice accessions were planted in single rows, and BRRI Dhan 28 was used as a check variety in every alternative row. The water level in the CF plots was maintained at a depth between 2 cm and 5 cm above the soil surface during the experiment. For AWD plots, a plastic pipe was used to monitor the water level. When the water level in the AWD plots reached 15 cm below the soil surface, the AWD plots were re‐irrigated between 2 cm and 5 cm above the soil surface. AWD irrigation was used from 14 days after transplanting until the initiation of flowering, at which points the AWD plots were managed the same as the CF plots. Straw and grain samples were hand harvested from the central six plants of each row when the grains had matured. The straw was hand harvested approximately 5 cm above the soil surface.

### 2.3 Analysis of Fe content in rice

Rice grains were oven‐dried at 80˚C for 3 days and de‐husked prior to analysis. To determine Fe concentration in rice gain, 0.2 g samples were weighed accurately out into 50 mL polyethylene centrifuge tubes followed by microwave digestion using nitric acid and hydrogen peroxide as described in Norton, Shafaei, et al., (2017). Similarly, straw samples were oven‐dried at 80˚C for 3 days and ball‐milled to a powder prior to analysis. The powdered samples (0.2g) were accurately weighed into a 50 ml polyethylene centrifuge tube and digested using nitric acid and hydrogen peroxide as described in Norton, Shafaei, et al. (2017). For each accession, there were 8 samples, four replicates of each rice accessions gown on the AWD plots and four grown on the CF plots. Analysis of Fe was done by inductively coupled plasma‐mass spectroscopy (ICP‐MS). Trace elements grade reagents were used for all digestions and certified reference materials (Oriental basma tobacco leaves [INCT‐OBTL‐5]) and rice flour [NIST1568b]), and blanks were used throughout the experiment as described in Norton, Shafaei, et al. (2017).

Analysis of Fe according to BAAP subgroups (Norton et al., 2018) was also conducted to examine whether the grain and straw Fe significantly differ within the BAAP subgroups.

### 2.4 GWA mapping of Fe content in rice

GWA mapping was performed using 2,053,863 SNPs derived from BAAP accessions (Norton et al., 2018). As described in Kang et al. (2010), the Parallel Identification of QTL’s using EMMAX (PIQUE) pipeline (https://github.com/tony‐travis/PIQUE) was used to perform GWA mapping of straw and grain Fe content using the previously curated BAAP SNP database (Norton et al., 2018). If any SNP has a minor allele frequency (MAF) <0.05, the SNP was excluded from the analysis and maximum per‐SNP missing was set at a 5% threshold. For SNP‐trait association, a mixed model was used across all accessions. In addition, a principal component analysis (PCA) was used to examine population structure which was used as covariates based on the first five principal components of the population. Using a kinship matrix, a random effect was estimated that allowed for the identification the genetic similarity in GWA analysis. To identify the false discovery rate (FDR) of SNP‐trait association, Benjamini‐Hochberg adjusted probabilities were estimated as described in Benjamini‐Hochberg (1995). A significant threshold of 10% FDR and *p *< 0.0001 were used to identify significant SNP (Norton et al., 2018).

Following GWA mapping of all traits, clump was used to bin together SNPs with *p *< 0.0001, MAF < 0.05 for all traits separately based on the linkage disequilibrium (LD) decay (Norton et al., 2019; Purcell et al., 2007). The global LD decay value of 243kb was used for CLUMP analysis as this is the average LD decay of the BAAP (Norton et al., 2018). Following the clump analysis, a QTL was defined on the basis of the criteria *p *< 0.0001 and a FDR<10%. If any SNP was identified *p *< 0.0001 but it does not meet the FDR criteria, it was defined as a putative QTL.

### 2.5 Downstream analysis of GWA mapping

Local LD decay was calculated based on r^2^ using PLINK within 1 Mbp region from the centre of a QTL (Norton et al., 2019; Purcell et al., 2007; VanLiere & Rosenberg, 2008). To identify potential candidate gene underneath the QTL, local LD decay was also used to confirm that the gene is within local LD decay of QTL region. To produce the LD heat map, R package LDheatmap was used, and QTL was visualised as local Manhattan plot on top of the LD heatmap. Mobile elements within LD were not considered as candidate genes. For all other genes underneath the QTL, the Rice Genome Annotation Project (RGAP) V7 annotation (Kawahara et al., 2013) and ARAMEMNON database (Schwacke et al., 2003) were examined for initial evidence of a functional link to the trait.

To identify synonymous and non‐synonymous SNPs within the interested genomic location, the 3,000 rice genome data (Mansueto et al., 2017) were obtained from the SNP‐Seek website. For the analysis of haplotype within a candidate gene for the BAAP, PLINK was used to extract SNPs data from ‘map’ and ‘ped’ files (Purcell et al., 2007). Subsequently, SNPs data and traits were aligned according to BAAP ID for the analysis. To identify whether any SNP or haplotype have an impact on trait, the effect size was determined by calculating mean and median, and data were visualised using box plots. The Haplotype Analysis tool in RiceVarMap2 was also used to investigate the haplotype of any gene in a larger population containing 4726 rice accessions (Zhao et al., 2015).

### 2.6 Statistical analysis

Statistical analyses were performed using R (version 3.6.1), Python (version 3.7.4), Minitab v.17 (State College, PA) and SigmaPlot v.14 (Systat Software Inc., CA). The normal distribution of residual assumption was checked before performing analysis of variance (ANOVA). Statistical significance was set at *p *< 0.05.

## 3 RESULTS

### 3.1 Straw and grain Fe content in rice

**FIGURE 1:**
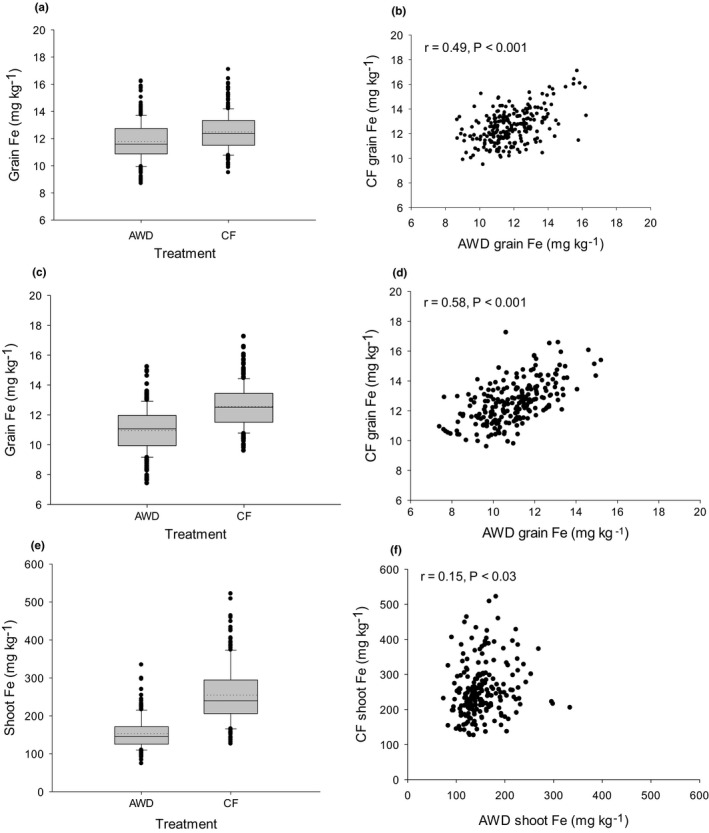
Box plots and scatter plots of Fe traits. Grain Fe (mg/kg) in AWD and CF in year 1 (a) and correlation between two traits (b). Grain Fe (mg/kg) in AWD and CF in year 2 (c) and the correlation between two traits (d). Straw Fe (mg/kg) in AWD and CF in year 1 (E) and correlation between two traits (f). In the box plot, the lower box boundaries (Q1) and upper box boundaries (Q3) represent 25 percentile and 75 percentile of data respectively. The height of the box is the interquartile range (IQR), and solid line inside the box indicates the median value. The length of whiskers below and above the box represents lower and upper values, respectively, outside the interquartile range but within 1.5 × IQR. The dots outside the whiskers represent lower and upper outliers (above 1.5 × IQR)

The mean and raw values for all straw and grain Fe traits obtained in this study are presented in Table S1. The AWD treatment significantly reduced the straw and grain Fe accumulation in the plants compared to the CF treatment, but the magnitude was much greater for straw (40%) compared to the grain (5.5–13%) (Figure 1, Tables 1 and 2). For grain Fe trait, the difference between the genotypes explained 35.4% and 34.8% of the observed variation in year 1 and year 2 respectively (Table 1). In year 1, there was a 1.9 (AWD)‐ and 1.8 (CF)‐fold difference in grain Fe between the accessions, while in year 2 a 2.1 (AWD)‐ and 1.8 (CF)‐fold variation in grain Fe was observed between the accessions (Table 2). For straw Fe trait, difference between genotype explained 16.1% of the observed variation (Table 1). A significant genotype by treatment interaction was observed for grain and straw Fe in year 1 but not in year 2 (Table 1).

**TABLE 1:** Descriptive statistics for grain and straw Fe concentration measured in 2013 and 2014 for the accessions grown under AWD and CF

Trait	Year		Descriptive statistics				
			Min	Max	Median	Mean	SD
Grain Fe ( mg/kg )	2013	AWD (*n* = 269)	8.7	16.2	11.7	11.8	1.4
		CF (*n* = 242)	9.5	17.1	12.4	12.5	1.4
Straw Fe ( mg/kg )	AWD (*n* = 260)	66.5	344.0	145.5	153.7	41.9
	CF (*n* = 218)	125.9	521.5	244.4	255.7	75.5
Grain Fe ( mg/kg )	2014	AWD (*n* = 244)	7.4	15.2	11.0	10.9	1.5
		CF (*n* = 225)	9.6	17.2	12.5	12.6	1.4

**TABLE 2:** Two‐way ANOVA results (F‐values) for treatment, genotype and the treatment x genotype interaction for the grain and straw Fe concentrations in 2013 and 2014. Numbers in brackets are the percentage contribution for that factor to the overall observed variation

Trait	Year	2‐way ANOVA		
		Treatment	Genotype	Treatment x genotype interaction
Grain Fe	2013	77.1 *** (3.0%)	3.8 *** (35.4%)	1.3 ** (11.7%)
Straw Fe		424.2*** (19.6%)	1.7 *** (16.1%)	1.3 ** (12.3%)
Grain Fe	2014	368.3 *** (13.3%)	4.4 *** (34.8%)	1.1 NS (8.8%)

^*^ *p* < 0.05, ** *p* < 0.01, *** *p* < 0.001, NS =not significant.

In each treatment, rice accessions were noted if they were in the top 10% of grain Fe concentration. There were twelve rice accessions identified which consistently showed high grain Fe across the experiments in both treatments (AWD and CF) (Table 3). Five accessions (Deshi boro, Jamir, Lakhai, Lahaya and Deshi boro) were in the top 10% grain Fe in both conditions for both years (Table 3), having on average grain Fe concentrations 22–34% higher than the population mean.

**TABLE 3:** Name and BAAP ID of rice accessions with consistently high grain Fe concentration in three or four of the experiments. An X indicates that the cultivar was in the highest ≈ 10% for grain Fe concentration in that experiment

BAAP ID	Cultivar name	BAAP subgroup/rice subpopulation	Year 1		Year 2	
			AWD	CF	AWD	CF
1	ASSAM 4 (BORO)	*aus* (Group 3)	X		X	X
100	ARC 14915	*aus admix*	X		X	X
110	Bir Cona	*aus* (Group 5)	X	X	X	
143	Jagal−1640 A	*aus* (Group 3)	X	X		X
165	Deshi Boro	*aus* (Group 3)	X	X	X	X
174	DA 2	*aus* (Group 3)	X	X	X	
222	Jamir	*aus* (Group 3)	X	X	X	X
231	Lakhai	*japonica*	X	X	X	X
234	Lahaya	*japonica*	X	X	X	X
235	Deshi boro	*indica*	X	X	X	X
238	Chhola boro (2)	*aus* (Group 3)	X	X		X
246	Gachi	*japonica*		X	X	X

Comparing traits between treatments revealed significant positive correlations which were much stronger for grain Fe than straw Fe (Figure 1). There was no significant correlation observed between shoot and grain Fe concentration under the two conditions (Figure S1). Examining the ratio of AWD/CF does not reveal any correlation between years for grain Fe or between grain and shoots in year 1.

### 3.2 Analysis of Fe in BAAP subgroups

**FIGURE 2:**
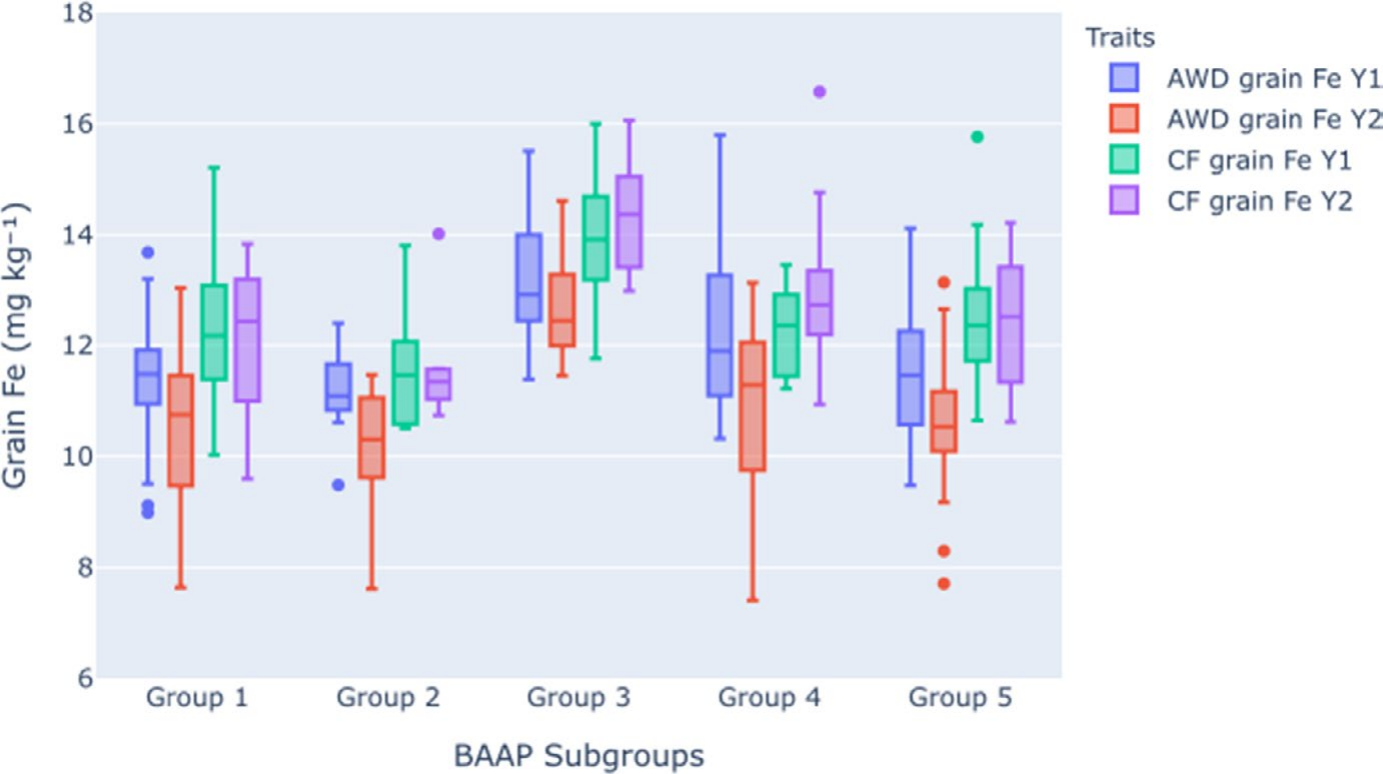
Variation of grain Fe (mg/kg) in BAAP subgroups at AWD and CF conditions. The frequency of BAAP subgroups is group 1 = 20, group 2 = 12, group 3 = 28, group 4 = 19 and group 5 = 29 accessions

A significant difference (*p *< 0.05) between BAAP subgroups was observed for all grain traits in AWD and CF conditions. For AWD grain Fe traits in year 1 and year 2, subgroups explained 30.0% and 33.4% of the variations respectively (Figure 2). For CF grain Fe year 1 and year 2 traits, subgroups explained 37.6% and 37.5% variations respectively (Figure 2). It was noticeable that group 3 had the highest mean and median grain Fe for all traits. The order (3>4>5>1>2) of mean grain Fe was found to be similar in all grain traits except CF grain Fe in year 1 which was 3>5>4>1>2. There was no significant effect observed for AWD straw Fe trait (Figure S2); however, a significant difference between subgroups was observed in the CF straw Fe trait (*p *< 0.01) where subgroups explained 14.8% variation (Figure S2). There was no difference between subgroup for the ratio of AWD/CF for grain Fe in either year or shoot Fe in year 1.

### 3.3 GWA mapping of straw and grain Fe content

**FIGURE 3:**
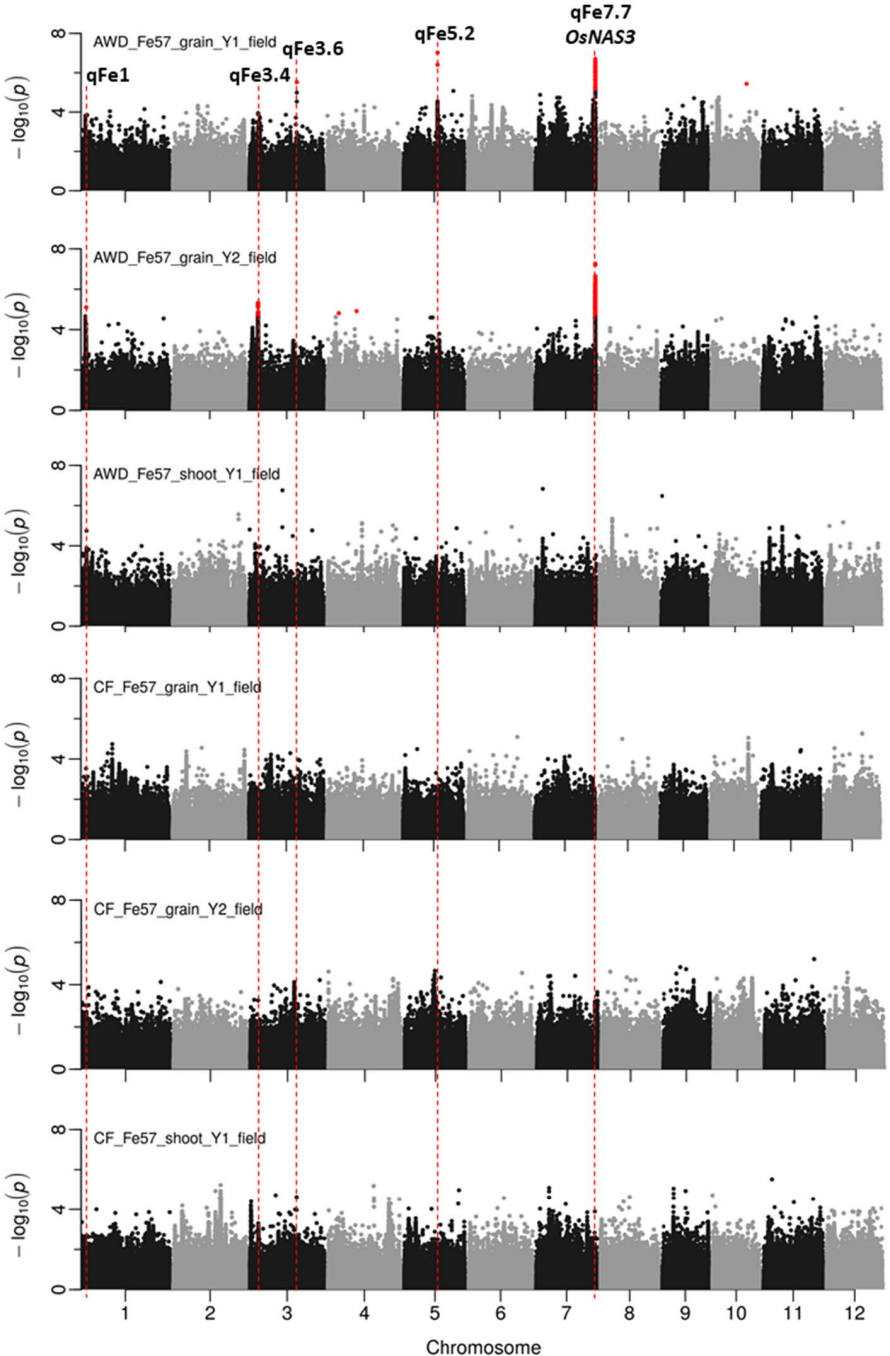
Manhattan plots for straw and grain Fe concentration when plants were grown under AWD or CF conditions. SNPs are positioned for 12 rice chromosomes along the x‐axis and y‐axis is the negative value of log of all SNPs associated with the p‐value. SNPs in red have a FDR <10%. For chromosome 1, 3, 5 and 7, the names and locations of QTLs are shown in the figure

Additional information about all BAAP accessions and value of all traits have been provided in the supplementary information (Table S1). The frequency distribution of the six traits used for GWA mapping is provided in Figure S3. GWA mapping was conducted on straw and grain Fe traits to identify genomic loci controlling natural variation of Fe accumulation in rice (Figure 3). Using GWA mapping, a total of 1388 SNPs (*p *< 0.0001) were identified as significantly associated for at least one of the six different straw and grain traits evaluated across the two years. Subsequently, CLUMP was used to identify single QTLs from multiple SNPs (*p *< 0.0001), and a total of 46 loci were identified considering the six traits (Table S2). Several clump groups overlapped (are within the global LD (243 kbp) of the BAAP); therefore, these were considered to be same putative QTLs (Table S2). A total of 40 QTLs were identified, which included two QTLs detected in two traits being at 29.3 Mb on chromosome 7 (for grain Fe in AWD both years) and at 18.7 Mbp on chromosome 10 (for grain Fe in CF both years). To reduce type one error, a Benjamini‐Hochberg false discovery rate of 10% FDR was applied. Of the 40 identified putative QTLs (Table S2), only 5 QTLs were found with at least one SNP above 10% FDR (Table 4). These 5 QTLs are discussed in detail below. Note, all of these QTLs were detected only in AWD conditions; however, a number of putative QTLs were detected under the CF conditions (Table S2).

**TABLE 4:** QTLs identified in this study (*p *< 0.0001, MAF < 0.05, FDR<10%) and their co‐localisation with previously identified QTLs for Fe content in rice

Traits	QTL name	Chromo‐some	Location (Mbp)	Co‐localisation with reported QTLs
AWD grain FeY2	qFe1	1	2.15	Yang et al. (2018)
AWD grain Fe Y2	qFe3.4	3	4.21	Norton et al. (2010) straw Fe; Swamy et al. (2018) grain Fe; Zhang et al. (2014) grain Fe.
AWD grain FeY1	qFe3.6	3	23.15	‐
AWD grain FeY1	qFe5.2	5	16.58	‐
AWD grain FeY1, Y2	qFe7.7	7	29.28	Anuradha et al. (2012); Descalsota et al. (2018); Zhang et al. (2014); Liu et al. (2020)

For AWD grain Fe year 1 trait, 3 different QTLs were identified on chromosome 3, 5 and 7. For AWD grain Fe year 2 trait, 3 QTLs were identified on chromosome 1, 3 and 7. There were no QTL (which made the FDR requirements) identified for the CF grain Fe trait, the CF straw Fe trait or the AWD straw Fe trait. Three of the five QTLs co‐localised with previous identified straw and grain Fe QTLs (Table 4).

### 3.4 QTL on chromosome 1 ~2.15 Mbp

A QTL (qFe1) was identified on top of chromosome 1 at 2.15 Mbp for AWD grain Fe year 2 trait (Figure 3, Table 4). The local LD decay of this region was found to be approximately 70 kbp (Figure S4). Only one significant SNP above 10% FDR threshold was identified within this QTL. There are 15 genes located within the local LD region of this QTL excluding ‘retrotransposon protein’ (Table S3).

### 3.5 QTLs on chromosome 3 ~4.21 Mbp and 23.15 Mbp

On chromosome 3, 2 QTLs were identified for AWD grain Fe trait. One of the QTLs (qFe3.4) was identified at 4.21 Mbp for AWD grain Fe in year 2 (Figure 3, Table 4). The local LD decay of this QTL region at 4.21 Mbp is approximately 120 kbp (Figure S4). A total of 13 SNPs significantly associated with the AWD grain Fe year 2 trait (*p *< 0.0001 and FDR <10%) in the QTL region. Within the local LD of this QTL (120 kbp), 41 genes are located excluding ‘retrotransposon protein’ (Table S4).

A QTL (qFe3.6) was also identified for AWD grain Fe trait in year 1 at 23.15 Mbp (Figure 3, Table 4). Only one significant SNP above 10% FDR threshold was identified within 1 Mbp genomic region of this QTL. In this QTL region, the local LD decay was approximately 590 kbp (Figure S4). Within the local LD of this, QTL 120 genes are located excluding ‘retrotransposon protein’ (Table S5).

### 3.6 QTL on chromosome 5 ~16.58 Mbp

A QTL (qFe5.2) was identified on chromosome 5 at 16.58 Mbp for AWD grain Fe year 1 trait (Figure 3, Table 4). Two significant SNPs above 10% FDR threshold were identified within 1 Mbp genomic region of this QTL. Local LD decay of this QTL is approximately 340 kbp (Figure S4). There are 69 genes and are located within this QTL region excluding ‘retrotransposon protein’ (Table S6).

### 3.7 QTL on chromosome 7 ~29.28 Mbp

**FIGURE 4:**
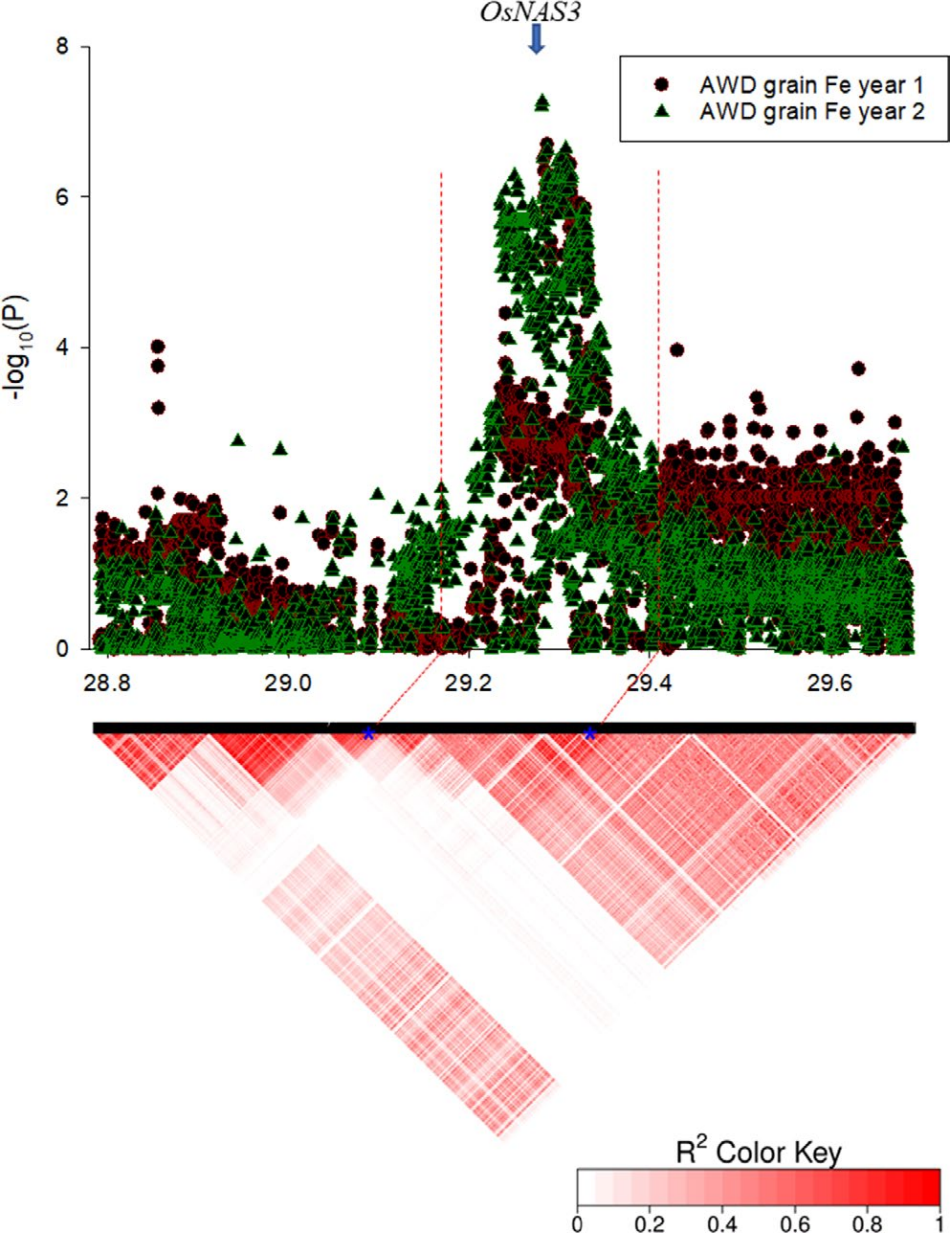
Local Manhattan plot (top) and LD heat map (bottom) produced within 1 Mbp region (28.80 Mbp to 29.80 Mbp) from the centre of the QTL (29.29 Mbp) on chromosome 7. The blue dots on the heat map shows the start of the QTL at 29.17 Mbp and the end of the QTL at 29.41 Mbp corresponding in the local Manhattan plot with red‐dotted vertical lines respectively. The blue arrow on the local Manhattan plot shows the location of *OsNAS3*

On chromosome 7, a QTL (qFe7.7) was identified at approximately 29.28 Mbp for grain Fe for the plants grown under AWD year 1 and year 2 (Figure 3, Table 4). A total of 134 and 357 SNPs within 1 Mbp region from the centre of the QTL (29.28 Mbp) were significantly associated with the traits (*p *< 0.0001 and FDR <10%) for year 1 and year 2 respectively. The local LD decay of this region was calculated to be 190 kbp (Figure S4). Within the local LD of this QTL, there are 54 genes excluding those genes annotated as ‘retrotransposon protein’ (Table S7). One of the genes is LOC_Os07g48980, annotated as ‘nicotianamine synthase, putative, expressed’. LOC_Os07g48980 is located from 29,323,094 to 29,324,723 bp (distance from the top of the chromosome) and was previously characterised as an enzyme *OsNAS3* (Figure 4). *OsNAS3* is responsible for DMA biosynthesis to solubilise Fe in soil and produce a Fe‐phytosiderophores complex which is taken up into root cells by YSL protein. To understand the natural variation of *OsNAS3*, SNPs within this enzyme of BAAP population were extracted using PLINK. It revealed the existence of four SNPs within *OsNAS3* (Table S8). Analysis of SNPs in 3,000 rice genomes also revealed that there are 4 common synonymous SNPs within *OsNAS3* in the *aus* subpopulation of rice which are also found in BAAP.

### 3.8 Haplotype analysis of *OsNAS3*

**FIGURE 5:**
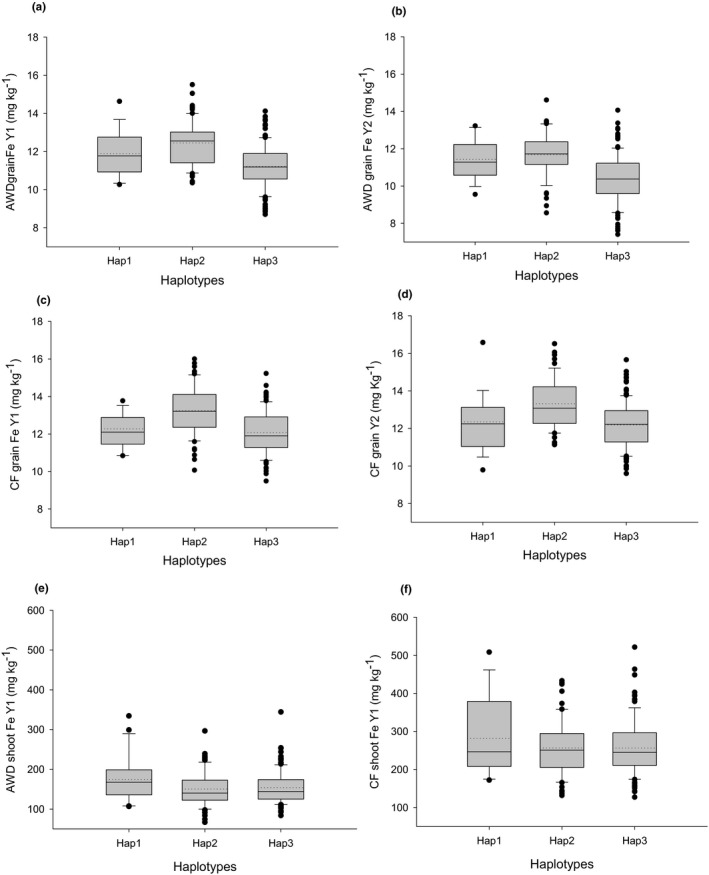
Box plots showing haplotypes‐traits association of *OsNAS3*. The allele frequency of haplotypes is haplotype 1 (*n* = 21), haplotype 2 (*n* = 74) and haplotype 3 (*n* = 143). Solid line within the box represents the median value, and the dotted line shows the mean value

Analysis of SNPs in the BAAP revealed three different haplotypes of *OsNAS3*. The plants with these haplotypes vary in Fe accumulation for plants grown under AWD and CF conditions in both years (Figure 5; Table S9). Analysis revealed that haplotype 2 of *OsNAS3* had the highest mean Fe across all grain traits in AWD and CF conditions. For straw Fe trait, haplotype 1 had the highest mean straw Fe in both conditions (Figure 5; Table S9). Haplotypes analyses of SNPs data using RiceVarMap2 derived from 4726 rice accessions indicated that there are 8 haplotypes of this gene across all rice subpopulations of which three have several *aus* accessions (I, II and V; Table S10). RiceVarMap2 haplotype I and VI share the same SNPs as BAAP haplotype 2, the high grain Fe allele, contributing to increase grain Fe up to 11%. Since RiceVarMap2 indicates no *aus* cultivars are haplotype VI, it suggests BAAP haplotype 2 is RiceVarMap2 haplotype I which is a *japonica*‐dominated haplotype (89% of *japonicas* have this haplotype cf. 4.8% of *indicas*). This allele therefore is rare in *indicas*, dominates *japonicas*, but it is well represented in *aus* (37% of *aus* have this allele). BAAP haplotype 1 is RiceVarMap2 haplotype V which is present in 16% of *aus* but is rare in *indicas* and *japonicas* (0.5% and 4.3% respectively). BAAP haplotype 3 matches RiceVarMap2 haplotype II, III and IV but is likely to be haplotype II since haplotype III and IV produced by RiceVarMap2 does not have any *aus* cultivars (Table S10). RiceVarMap2 haplotype II is an *indica*‐dominated haplotype with 88% of *indicas* having this allele, but only 7% of *japonicas* and 47% of *aus*. The distribution of haplotypes between *indicas* and *japonicas* described above clearly show that it is only in *aus* where allele frequency is reasonably balanced, enhancing the chances to detect effects in GWA mapping. When the BAAP subgroups and BAAP haplotypes of *OsNAS3* are analysed together, it was observed that all of the 28 accessions from BAAP subgroup 3 are in BAAP haplotype 2 (Table S8). This demonstrates that BAAP subgroups 3 contains important allele to improve grain Fe in rice.

For straw Fe trait, BAAP haplotype 1 contributed to higher mean straw Fe regardless of AWD or CF condition (Figure 5; Table S9). RiceVarMap2 data revealed that BAAP haplotype 1 is not common in the two major sub‐species (*Indica*, *Japonica*) as well as in *aus* subpopulation of rice. However, it was found that *aus* has the highest frequency of BAAP haplotype 1 among three subpopulations of rice (*indica*—0.5%, *japonica*—4.4%, *aus*—16%) (Table S10).

## 4 DISCUSSION

### 4.1 Fe content in AWD and CF condition

This study investigated Fe accumulation in a rice population (BAAP) grown under two different irrigation systems. Analysis of straw and grain Fe of the BAAP accessions indicated that AWD reduced straw and grain Fe accumulation in rice. A study conducted by Norton, Travis, et al. (2017) on 22 rice accessions reported that AWD reduced grain and straw Fe by 5.8% and 12.9% respectively. This study was conducted on a larger population for multiple years where it was observed that AWD reduced mean grain Fe 5.5% and 12.9% in year 1 and year 2 respectively. It was also observed that AWD reduced straw Fe 39.9%. The previous study mentioned that in aerobic and anaerobic conditions, soil chemistry play an important role to oxidise and reduce soil respectively (Zhang et al., 2014). It was assumed that soil chemistry had an impact on straw and grain Fe accumulation in rice in AWD and CF condition.

It was noted that there was a significant genotype by treatment interaction in year 1 which explained 11.1% variation for grain Fe. There was no significant interaction observed for grain Fe trait in year 2. This suggests that while genotypes might differ slightly in relative grain Fe depending on irrigation management, breeding for high grain Fe need not be done specifically for AWD. This study has identified 12 rice accessions which consistently had high grain Fe in AWD and CF conditions over two‐year period (Table 3) with approximately 30% more grain Fe than the population mean. It implies that breeding could be useful strategy to develop high grain Fe accumulating rice regardless of AWD or CF condition.

### 4.2 Identification of co‐localised Fe regulatory QTLs

QTL mapping to identify natural variation of genomic location controlling grain Fe using biparental mapping populations has been conducted in a number of studies (Anuradha et al., 2012; Calayugan et al., 2020; Dixit et al., 2019; Jeong, Bombay, et al., 2020; Jeong, Lee, et al., 2020; Norton et al., 2010; Swamy et al., 2018; Zhang et al., 2014), and to date, six GWA studies have been conducted to identify natural variation of grain Fe in rice (Bollinedi et al., 2020; Descalsota et al., 2018; Liu et al., 2020; Pradhan et al., 2020; Yang et al., 2018; Zaw et al., 2019). This study has been conducted for multiple years in two different conditions (eg AWD, CF) with a subpopulation of rice, *aus*, which is known to contain several good traits in rice including disease resistance (Garris et al., 2003), drought tolerance (Bernier et al., 2009), low phosphorous tolerance (Gamuyao et al., 2012), submergence tolerance (Hattori et al., 2009; Xu et al., 2006) and is known as an unique resource of plant breeders to develop climate‐resilient rice varieties (Kim et al., 2016). In this study, 40 putative QTLs were detected using BAAP, mainly consisted of *aus* subpopulation of rice, of which five passed the 10% FDR threshold. It is noteworthy that only a small number of significant QTLs identified in this study and there was no significant QTL identified for CF grain Fe in both year 1 and year 2 as well as in both AWD and CF straw Fe trait. It could be due to the approach used for the identification of QTL (eg 10% FDR value and *p *< 0.0001). This stringent approach has been also used in similar studies before to avoid false discovery of SNPs in GWA studies (Chen et al., 2020; McCouch et al., 2016), but the 35 putative QTLs detected in the current study may yet prove to be real loci regulating rice Fe. Three of the five significant QTLs identified in this study, co‐localised with previously identified studies, are discussed below as these QTLs considered to be more reliable (Table 4).

The QTL (qFe1) identified on chromosome 1 at 2.15 Mbp for AWD grain Fe in year 2 was previously identified by Yang et al. (2018). The LD decay of this QTL in BAAP was found to be 70 kbp which is comparatively lower than global LD decay (243 kbp) and LD decay (245 kbp) of chromosome 1 of this population (Norton et al., 2018). It demonstrates that the local LD of BAAP population differs across the chromosomes. It could be due to the fact that this QTL region is a hotspot for high recombination which breaks down the haplotype blocks and results in lower LD of this genomic region. Due to lower LD decay, only 15 genes were identified within this QTL region (Table S3). However, there is no gene identified within this QTL region associated with iron transport in rice. As this QTL has been identified for multiple studies using different population, it might be a potential QTL to consider for grain Fe in rice.

One of the QTLs identified in this study is on chromosome 3 at 4.21 Mbp (qFe3.4). A study conducted by Norton et al. (2010) identified a leaf Fe QTL around 4 Mbp on chromosome 3 using a biparental mapping population. A different study conducted by Swamy et al. (2018) also identified QTL for grain Fe trait near to the same genomic region using two *O*. *nivara* derived back‐cross populations. In addition, Zhang et al. (2014) also reported a QTL for grain Fe trait near to the same genomic region where a biparental back‐cross population was used for QTL mapping. There were 41 genes identified within this QTL region and one of the genes, LOC_Os03g08070, is annotated as a heavy metal‐associated protein (HMP) (Kawahara et al., 2013). LOC_Os03g08070 is categorised as a P_1B_‐ATPase (Schwacke et al., 2003). P_1B_‐ATPase has been reported to act as a Fe efflux pump in bacteria (Turner et al., 2017). Although speculative, it is an interesting possibility that LOC_Os03g08070 might transport Fe and should be tested using gene editing.

In addition to the QTLs on chromosome 3, a QTL (q7Fe) on the bottom of chromosome 7 was also identified for AWD grain Fe trait in year 1 and year 2. This QTL has been also previously identified for grain Fe traits in different populations (Anuradha et al., 2012; Descalsota et al., 2018; Liu et al., 2020; Zhang et al., 2014). The identification of this QTL in multiple years for plants grown under AWD condition using BAAP and co‐localisation of this QTL with several previous studies suggests that this is a stable QTL which could be used to improve grain Fe in rice.

### 4.3 Natural variation of q7Fe and *OsNAS3*

One of the enzymes located within q7Fe QTL is *OsNAS3* characterised as nicotianamine synthase enzymes. Previous studies have demonstrated the important role of nicotianamine synthase enzymes to improve grain Fe accumulation in rice (Aung et al., 2019; Descalsota et al., 2018; Inoue et al., 2003; Kobayashi et al., 2014; Moreno‐Moyano et al., 2016; Trijatmiko et al., 2016). Three nicotianamine synthase (NAS) enzymes (*OsNAS1*, *OsNAS2* and *OsNAS3*) have been identified in rice (Inoue et al., 2003). A previous study demonstrated the constitutive overexpression of *OsNAS1*, *OsNAS2* and *OsNAS3* increases 2.4‐, 3.5‐, and 2.7‐fold grain Fe in rice respectively (Johnson et al., 2011). *OsNAS2* has been used to produce high grain Fe in transgenic plants for future release to farmers (Trijatmiko et al., 2016), but *OsNAS3* has not been targeted. A recent study has identified a twofold difference of *OsNAS3* expression in rice grain within 3 rice genotypes (Descalsota et al., 2018) indicating that genotypic differences in gene expression exist for *OsNAS3* and therefore might be associated with increased grain Fe phenotype in rice.

Three haplotypes of *OsNAS3* with the 3K rice database were described where a rare allele with only 30 accessions (1.3%) was high in Fe and Zn (Abbai et al., 2019). The use of BAAP also identified three haplotypes of *OsNAS3* in this study (Table S8). One of the important findings of this study is that the frequency of high grain Fe allele identified in BAAP is 31%. Obviously, the frequency of this high Fe allele in *aus* suggests that it would be easier to detect the QTL and its impact in an *aus* panel than in an indica panel where it is rare, or a *japonica* panel where the high grain Fe allele is dominant, or in a mixture of the any two panels where population structure will hinder its detection. This information will help breeders to choose new parent cultivars to develop new varieties. For plant breeders, *aus* is an important resource to develop new rice varieties (Kim et al., 2016). The identification of high allele frequency of *OsNAS3* in BAAP further indicates the importance of *aus* in rice breeding.

### 4.4 Linked between BAAP subgroups and haplotypes

The analysis of all grain Fe traits in AWD and CF conditions revealed that BAAP subgroup 3 contributed for the highest mean grain Fe in AWD and CF conditions. It was also notable that BAAP subgroup 3 contributed high mean grain Fe for haplotype 2 of *OsNAS3* discussed in this study. For example, all of the accessions in BAAP subgroup 3 have BAAP haplotype 2 of *OsNAS3* (Table S8, Figure 5). Of the 12 cultivars identified as high in grain Fe presented in Table 3, six are in subgroup 3, one is in subgroup 5, one is an *aus*‐admix of subgroup 1 and 5, while two are *japonicas*, one is an *indica* and one is unknown. Subgroup 3 can be used to improve grain Fe in rice using conventional plant breeding. Further phylo‐geographic information that explains the origin of subgroup 3, which predominantly come from Bangladesh, would be interesting.

## 5 CONCLUSION

This study has improved the current knowledge on Fe accumulation in rice in AWD and CF conditions. Although AWD has reduced overall grain Fe accumulation, a number of rice accessions have been identified in BAAP which consistently had high grain Fe in both AWD and CF. The finding of these high grain Fe accumulating rice accessions in different conditions as well as knowledge on BAAP subgroup 3 (which has high grain Fe) will allow breeders to add these accessions in the breeding programme to develop varieties with improved grain Fe. In addition, this study has reported five QTLs controlling grain Fe in AWD condition for the first time. From these, a candidate gene, *OsNAS3*, has been identified within the QTL controlling natural variation of grain Fe accumulation. The findings of the SNPs markers and haplotypes in *OsNAS3* will provide useful information for breeders to increase grain Fe in rice.

## AUTHOR CONTRIBUTIONS

GJN and AHP involved in conception and funding acquisition. MHS and MRI involved in managing field experiments. PT, GJN, AJT and AHP analysed the data and conducted bioinformatics. PT, GJN and AHP wrote the manuscript. All authors have read and approved the manuscript.

## Supporting information

Fig S1‐S4

Table S1

Table S2

Table S3

Table S4

Table S5

Table S6

Table S7
