# Supplementary material for: Identification of genomic loci regulating grain iron content in aus rice under two irrigation management systems
Source: Food Energy Secur. 2021 Sep 30;11(1):e329. doi: 10.1002/fes3.329 (PMC9286631; doi:10.1002/fes3.329)
Supplement: Supplementary file 1 — Fig S1‐S4 [file FES3-11-0-s005.docx]

**Supplementary Figures:**

**Supplementary Figure S1.** Correlation between Shoot and grain Fe (mg kg^-1^) in AWD (A) and CF (B) conditions**.**

**Supplementary Figure S2.** Variation of shoot Fe (mg kg^-1^) in BAAP subgroups at AWD and CF condition in year 1.


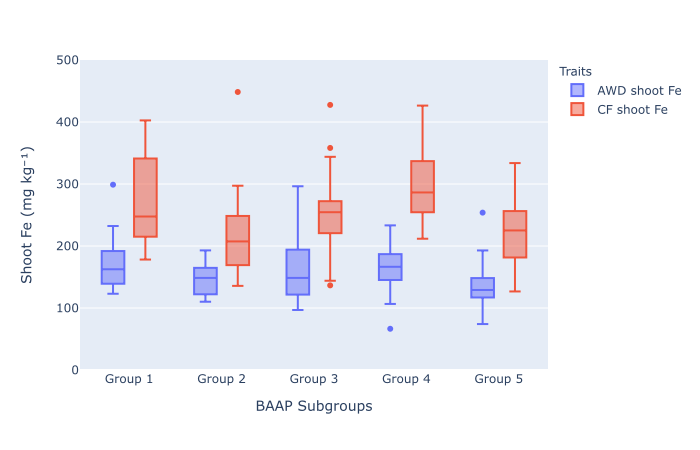


**Supplementary Figure S3.** Frequency distribution of six traits used for GWA mapping of Fe content in rice. X axis denotes the name of traits and y axis denotes the number of rice accessions.


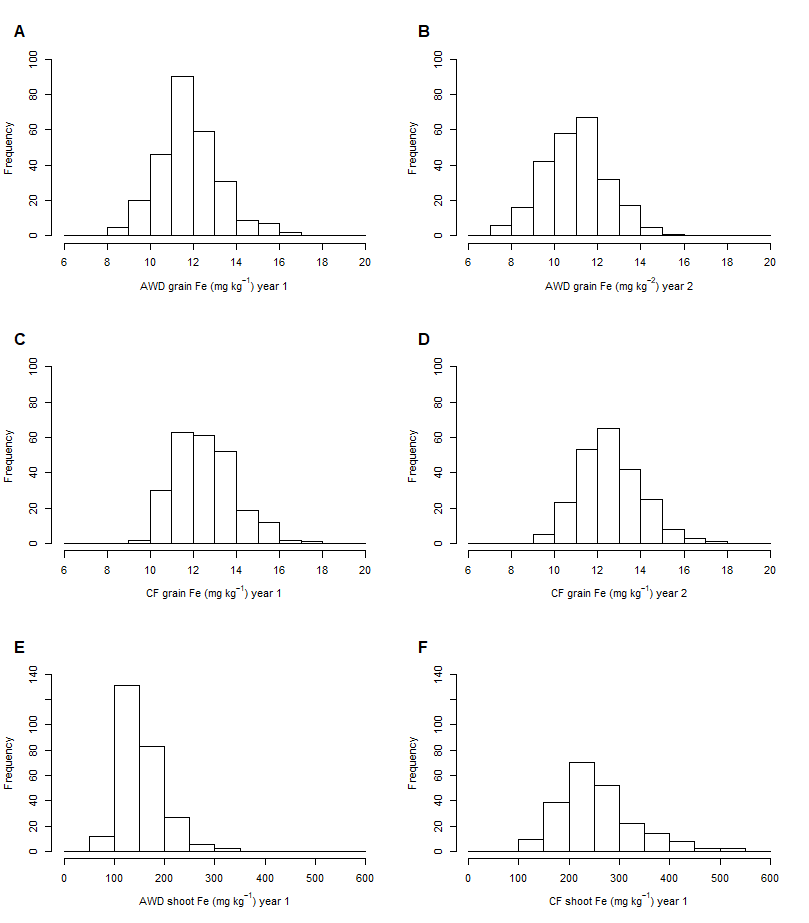


**Supplementary Figure S4.** Local R^2^ within 1 Mbp of QTLs on chromosome 1, 3, 5 and 7.
